# Trauma Burden Affected People with Multiple Sclerosis During SARS-CoV-2 Pandemic

**DOI:** 10.3390/jcm14082665

**Published:** 2025-04-13

**Authors:** Felicita Heidler, Michael Hecker, Niklas Frahm, Julia Baldt, Barbara Streckenbach, Janina Meißner, Katja Burian, Silvan Elias Langhorst, Pegah Mashhadiakbar, Jörg Richter, Uwe Klaus Zettl

**Affiliations:** 1 Department of Neurology, Ecumenic Hainich Hospital gGmbH, 99974 Mühlhausen, Germany; f.heidler@oehk.de (F.H.); j.meissner@oehk.de (J.M.);; 2 Neuroimmunology Section, Department of Neurology, Rostock University Medical Center, 18147 Rostock, Germany; michael.hecker@med.uni-rostock.de (M.H.); uwe.zettl@med.uni-rostock.de (U.K.Z.); 3 Faculty of Health Sciences, University of Hull, Hull HU6 7RX, UK

**Keywords:** trauma, multiple sclerosis, SARS-CoV-2, pandemic, anxiety, depression, vaccination willingness, polypharmacy, multimorbidity

## Abstract

**Background/Objectives:** Trauma is a psychological injury resulting from a distressing or overwhelming event. The SARS-CoV-2 pandemic has been disruptive and traumatic for many people with multiple sclerosis (pwMS). The relationship between patient characteristics and trauma in pwMS during the pandemic has not yet been thoroughly explored. The aim of this bicentric prospective cohort study was to analyze the prevalence and development of probable post-traumatic stress disorder (PTSD) among pwMS during the SARS-CoV-2 pandemic and to identify patient parameters associated with this condition. **Methods:** We have assessed pwMS for probable PTSD before and after the approval of the first SARS-CoV-2 vaccines in Germany using an adapted version of the Trauma Screening Questionnaire (TSQ). We compared pwMS with probable PTSD (TSQ sum score ≥ 6) with those without probable PTSD (TSQ sum score < 6) regarding sociodemographic and MS-specific clinical characteristics, polypharmacy status, comorbidities, anxiety/depression levels, personality traits, mental/physical/social burden, and general vaccination willingness. **Results:** Out of the 149 pwMS included, 8.1% were identified as having probable PTSD. These patients had significantly higher rates of pre-pandemic abnormal anxiety (66.7% vs. 18.5%, *p* < 0.001) and depression scores (45.5% vs. 12.6%, *p* = 0.013). The patients with probable PTSD exhibited a distinct personality profile, with significantly higher neuroticism and harm avoidance scores and lower conscientiousness, cooperativeness, and self-directedness scores. They were also significantly more likely to report severe or very severe mental/physical/social burdens during the pandemic compared to those without probable PTSD (*p* ≤ 0.045). **Conclusions:** Medical and social services should be provided to support patients who experienced serious stress or trauma. The development of concepts for education and vaccination procedures should be accompanied by comprehensive and clear communication that recognizes individual risk factors and addresses possible concerns with evidence-based and convincing arguments.

## 1. Introduction

Trauma is a profound experience in the life of a person, which means a psychologically exceptional situation for the respective person [1]. Trauma usually refers to a psychological or physical injury caused by a stressful or upsetting event. According to the currently applied international psychiatric classifications (ICD-11, DSM-V), a traumatic event is defined as exposure to death, threatened death, actual or threatened serious injury, or actual or threatened sexual violence [1,2]. Such events can cause strong feelings of powerlessness or loss of control, disrupted beliefs and expectations, serious questioning of basic assumptions about life, and profound disturbance in self-trust and trust in others or the certainties of life [2]. According to a comprehensive report on World Mental Health [3], 70% of survey respondents from the general population reported exposure to a traumatic event. Studies have indicated that a significant proportion of people in developed countries have been exposed to at least one traumatic event in their lifetime. A life event associated with post-traumatic stress disorder (PTSD) is a traumatic or extremely stressful event that a person has experienced, which may have triggered or been associated with the development of PTSD [4]. The most common events reported are the unexpected death of a loved one and motor vehicle accidents [3]. While most individuals exposed to trauma demonstrate resilience, a significant minority develop PTSD, which is a mental disorder associated with trauma. The lifetime prevalence of PTSD in high-income countries ranges from 1.3% (Japan) to 8.8% (Northern Ireland) [5]. Improper handling of a traumatic event due to various risk factors can lead to the development of somatic and psychological symptoms, which may result in PTSD.

Trauma can manifest in various symptoms, and individuals may experience them to different degrees. Symptoms of PTSD include repeated and intrusive memories of an overwhelmingly traumatic event, avoidance of stimuli associated with the traumatic event, nightmares, and flashbacks [1,6]. The reminders last more than 1 month and start within 6 months of the event [6]. People may avoid situations, places, or activities that remind them of the trauma, which can lead to social withdrawal and a desire to isolate themselves from others. Trauma can lead to negative changes in thinking patterns and emotions like negative beliefs about themselves or the world, feelings of guilt or shame, difficulty experiencing positive emotions, and a sense of detachment from others. People may have changes in emotional reactivity, such as heightened anxiety, exaggerated emotional responses to stimuli, irritability, and difficulty concentrating. Hypervigilance is also common, where individuals remain excessively alert and vigilant, constantly scanning their environment for potential threats. This state of hypervigilance can cause feelings of anxiety and prevent relaxation. Trauma can manifest as physical symptoms such as headaches, stomachaches, and other unexplained pains. These symptoms may not have a clear medical explanation [1,7]. PTSD can result in significant interpersonal and occupational difficulties. According to Kessler [8], it may lead to an estimated loss of 3.6 days of productivity per month. Due to its long-term effects, PTSD has been referred to as a “life sentence”. Studies have shown that an increased risk of chronic diseases, accelerated aging, and premature mortality are associated with the factors mentioned [9,10,11,12]. It is important to note that responses to trauma can vary greatly among individuals and that not all who experience a traumatic event will develop symptoms.

Several studies have highlighted the exceptional nature of the pandemic of the severe acute respiratory syndrome coronavirus 2 (SARS-CoV-2) and the mental [13,14] and physical toll [12] that the fear of infection, isolation, and its consequences have had on those affected. In the coronavirus disease 2019 (COVID-19) dashboard at the World Health Organization (WHO), over 777 million confirmed cases [15] and over 7 million deaths [16] by or with COVID-19 have been reported by April 2025. The pandemic presented a life-threatening situation, particularly for people with multiple sclerosis (pwMS) [13,17,18,19,20,21,22], whose immune systems are weakened [23,24,25,26,27]. MS is a neuroimmunological disorder of the central nervous system [28,29]. It is the most common chronic neurological disease in young adults and affects over 2.8 million people worldwide [30]. MS symptoms include a wide range of neurological deficits, such as acute unilateral optic neuritis, diplopia, hyposensitivity, dysesthesia, nystagmus, trigeminal neuralgia, motor dysfunction, cerebellar ataxia, bladder dysfunction, fatigue, depression, and neuropsychological deficits [31,32]. The early age of MS onset and the significant impact on life quality and productivity cause a considerable disease burden [33]. Compared to the general population, pwMS have a significantly higher risk of developing infections, including bacterial, viral, and fungal infections [26,34,35,36]. Infections are a leading cause of death in pwMS. A study of US veterans found that the overall mortality rate among pwMS was 2.9 times higher than that of the general population. The infection mortality rate is increased by a factor of 6.2 [37]. The weakened immune system of pwMS is attributed to both the patients’ underlying MS disease and the use of disease-modifying therapies (DMTs) [36,38,39,40,41,42]. Regarding some high-efficacy DMTs used to treat MS, particularly early in the pandemic, it was not clear whether they significantly increased the risk of SARS-CoV-2 infection in pwMS. This uncertainty led to discussions about whether the use of these drugs should be stopped [43,44]. In addition, many MS outpatient clinics closed temporarily or offered only reduced consultation hours to minimize the risk of SARS-CoV-2 infection for patients and employees and to increase the availability of doctors in the acute treatment of patients. This situation caused significant distress and could have been a profound source of traumatic burden in pwMS.

Despite existing research on PTSD and MS [45,46,47], there is a lack of studies specifically examining the psychological impact of the SARS-CoV-2 pandemic on pwMS. Recognizing the need for further research, we set out to explore the trauma burden in pwMS during the SARS-CoV-2 pandemic. To our knowledge, it is the first study that longitudinally evaluated traumatic stress in pwMS at two points in time during the pandemic. We hypothesized that, given the extraordinary circumstances of the pandemic, trauma-related symptoms would be a critical issue for pwMS and that investigating their frequency, persistence, and relationships with other psychometric assessments would reveal important insights into the impact of the pandemic on this population.

In this study, we analyzed the prevalence of probable PTSD among pwMS during the SARS-CoV-2 pandemic at two time points: approximately 3 months after the WHO declared the COVID-19 outbreak a global pandemic and 1 year after the approval of the first SARS-CoV-2 vaccines in Germany. Patients with and without probable PTSD were compared regarding sociodemographic and MS-specific neurological characteristics, polypharmacy, comorbidities, anxiety/depression levels, mental/physical/social burden, and general vaccination willingness. By this means, we aimed to explore the associations of probable PTSD with pre-existing anxiety/depression, personality traits, and the overall psychosocial burden experienced during the pandemic. The insights from this study are valuable for developing targeted interventions and support strategies to improve the well-being of pwMS in future health crises.

## 2. Methods

### 2.1. Study Population and Inclusion Criteria

This prospective cohort study was conducted at the Department of Neurology (Neuroimmunology Section) at the Rostock University Medical Center (Germany) and at the Department of Neurology of the Ecumenical Hainich Hospital, Mühlhausen (Germany). The patients were primarily of Western European ancestry. They were enrolled consecutively as they presented at the clinic. To be included in the study, the patients had to be 18 years of age or older. Moreover, only patients who had a clinically isolated syndrome (CIS) or who fulfilled the 2017 revised McDonald criteria for the diagnosis of MS were included [48]. Participation in this study was voluntary, and each patient was assigned a participant number after providing written informed consent. Exclusion criteria were unwillingness to participate or a poor cognitive state that hindered study participation. To ensure pseudonymization, interview sheets and questionnaires were labeled with numbers instead of names or other identifying information.

### 2.2. Data Acquisition

For this longitudinal study, the data collection was carried out over three time periods. Baseline data were gathered between June 2019 and June 2020. For this purpose, pwMS were interviewed either after their medical appointments as outpatients or on the neurological ward as inpatients. Baseline data comprised sociodemographic, clinical, and therapeutic characteristics of pwMS as well as willingness to receive recommended standard vaccinations. Follow-up interviews were conducted approximately 3 months after the WHO declared the COVID-19 outbreak a pandemic (first follow-up: May to July 2020) and approximately 1 year after the authorization of the first SARS-CoV-2 vaccines in Germany (second follow-up: October 2021 to January 2022) to assess disease activity (relapses/progression) and pandemic-associated traumatic, mental, physical and social burden.

#### 2.2.1. Baseline Data

Sociodemographic information (e.g., age and sex), MS-related clinical data (e.g., years since diagnosis, MS course type, and Expanded Disability Status Scale (EDSS) score [49]), as well as data on comorbidities and medications, were collected through a structured interview, medical records and clinical examination. During the standardized interview, which lasted approximately 40 min, the patients were asked the same set of questions in the same order. The EDSS score measures the level of disability in pwMS. The scale ranges from 0 to 10 in 0.5-unit increments. A score of 0 indicates no deficits, while a score of 10 indicates death due to MS [49]. Disease duration was calculated based on the date of CIS/MS diagnosis.

The NEO-Five Factor Inventory (NEO-FFI) was the first questionnaire used to assess personality characteristics among pwMS in our study [50,51,52]. It comprises 60 items with a 5-point Likert-type answer model (ranging from “strongly disagree” to “strongly agree”) and assesses five factors: neuroticism, extraversion, openness, conscientiousness, and agreeableness. The total number of items for each of the 5 factors is 12. In this analysis, each item was assigned a value ranging from 0 to 4, depending on the selected answer on the 5-point Likert scale. The scores for the 5 factors can be interpreted in accordance with the established methodology. For instance, high scores for neuroticism may indicate emotional instability, anxiety, fear, nervousness, and a tendency toward excessive worry or rumination. High scores for extraversion may suggest activity, cheerfulness, confidence, sociability, and assertiveness. High scores for openness may indicate imagination, intellectual curiosity, a preference for novelty, and a willingness to explore new experiences. High scores for conscientiousness may indicate determination, diligence, perseverance, reliability, and strong goal-directed behavior. High scores for agreeableness may indicate cooperativeness, interpersonal trust, empathy, and a preference for social harmony [53]. Schwartz et al. investigated the use of the NEO-FFI in pwMS and found it to be appropriate [54]. They reported satisfactory internal consistency (with Cronbach’s α ranging from 0.71 to 0.87, depending on the personality trait), factorial validity (with scores from 0.82 to 0.87), and congruence between self- and other reports (with Pearson correlation coefficients ≥ 0.33) [54].

We also utilized the Temperament and Character Inventory-Revised (TCI-R) to assess personality characteristics [55,56,57]. The German version of the TCI-R requires respondents to answer 240 items using a true/false answer model. Temperament dimensions (novelty seeking, harm avoidance, reward dependence, and persistence) are relatively stable over time and represent automatic emotional responses. Character dimensions (self-directedness, cooperativeness, and self-transcendence) capture individual differences in values and norms. Analyses demonstrated the factorial, convergent, and discriminant validity of the TCI-R [58]. Its content validity has been supported by the correlation structure with the Gießen test, an instrument to assess social self-perception [58]. Among a general population sample, the internal consistency was satisfactory for all TCI-R dimensions (Cronbach α coefficients: 0.71–0.87). In factor analyses, the cumulative variances for temperament and character were 58.1% and 59.6%, respectively [59]. For interpretation of the temperament dimensions, individuals with high scores for novelty seeking are likely to exhibit exploratory behavior, impulsiveness, excitability, and a tendency to seek new and stimulating experiences. High harm avoidance is associated with pessimism, shyness, anxiety, fear of uncertainty, and excessive worry. People with high scores for reward dependence may tend to be warm-hearted, sensitive, and socially attached. Finally, individuals with high scores for persistence may tend to be diligent, persevering, and determined. In the context of character dimensions, people with high scores for self-directedness may exhibit maturity, purposefulness, and reliability, while those with high scores for cooperativeness may demonstrate consideration, friendliness, and tolerance. Moreover, individuals who exhibit elevated levels of self-transcendence are more likely to possess qualities such as humility, patience, selflessness, and a sense of unity with something greater than themselves [53].

The Hospital Anxiety and Depression Scale (HADS) is a valid screening tool with suitable psychometric properties for diagnosing anxiety (HADS-A) and depressive symptoms (HADS-D) in pwMS [60]. The HADS comprises 14 symptom items, seven for measuring anxiety and seven for depression, which are answered on a four-point response model (0–3). A clear two-factor structure was found in factor analysis, and substantial correlations with the Beck Depression Inventory, the State–Trait Anxiety Inventory, and domain scores of the Short-Form Health Survey indicated its high concurrent validity in a study of its construct validity [61]. Marrie et al. reported good test-retest reliability (intraclass correlation coefficient: 0.83) and good internal consistency (Cronbach’s α = 0.82) for HADS-D as well as good test-retest reliability (0.83) and internal consistency (Cronbach’s α = 0.86) for HADS-A in pwMS [62]. Generally, normal HADS scores range from 0 to 7 points, borderline scores range from 8 to 10 points, and abnormal scores range from 11 to 21 points [62]. Applying a cut-off point of ≥8 effectively detects diagnoses of current major depression and generalized anxiety disorder in pwMS, with HADS-A showing 82% sensitivity and 68% specificity and HADS-D showing 69% sensitivity and 81% specificity [62].

#### 2.2.2. Follow-Up Data During the SARS-CoV-2 Pandemic

At both follow-up visits, the pwMS were asked to complete an adapted version of the Trauma Screening Questionnaire (TSQ). The TSQ is a screener for PTSD [63], consisting of a 10-item self-report scale derived from the 17-item PTSD Symptom Scale [64,65,66]. The TSQ comprises 10 items, 5 related to arousal and 5 to re-experiencing symptoms. For the purpose of the present study, we modified the TSQ to assess the impact of the SARS-CoV-2 pandemic and the associated information and restrictive social measures. This was performed by specifically adapting the wording of the 10 individual items to the context of the SARS-CoV-2 pandemic. We also modified the original response model to a 5-point scale ranging from 0 (not at all) to 4 (very strongly) to provide a more differentiated assessment of the traumatic burden with regard to the SARS-CoV-2 pandemic (see detailed items in Supplementary Table S1). Galea et al. employed the TSQ with a 5-point response scale as well [67]. To assess the burden associated with in-country SARS-CoV-2 infection control measures and living with the risk of infection and severe COVID-19 comorbidity in addition to present MS, a sum score of the 10 symptom items was calculated by reclassification of the 5-point scale into a 2-point scale (0/no: not at all, almost not, not sure; 1/yes: strongly, very strongly), as shown in Supplementary Table S2. A total score of 6 or greater on the TSQ is indicative of an elevated risk of PTSD, as evidenced by different studies [64,68,69,70]. The reclassification of a 5-point scale into a 2-point scale was also employed in the study by Irizar et al. on associations of probable PTSD and alcohol abuse in military and police personnel [68]. However, Irizar et al. classified responses other than “not at all” as 1/yes, reflecting an optimistic estimation of the prevalence of probable PTSD. Conversely, we opted for a more conservative estimation, as it is crucial to highlight that a score of 6 or above on the adapted TSQ may indicate a trend but should not be interpreted as a definitive diagnostic criterion for PTSD [68,70,71]. In our study, Cronbach’s α for the adapted TSQ was 0.863, indicating a high internal consistency. Additionally, the patients were asked whether they had experienced an MS relapse or progression in the three months prior to the surveys. Moreover, the occurrence of perceived mental, physical, and social stress during the SARS-CoV-2 pandemic was rated by the pwMS on a scale from “not at all” to “very severe”. The follow-up questionnaires took up to 15 min to complete.

### 2.3. Statistics

Statistical analyses and data visualizations were performed using SPSS, version 27, and R, version 4.1.2. Pre-pandemic baseline data on sociodemographic and MS-specific neurological characteristics, presence of polypharmacy (concurrent use of at least 5 drugs), number of comorbidities, presence of psychiatric comorbidities, smoking history (ever smoked: yes/no), HADS score levels, NEO-FFI scores, TCI-R scores, and general vaccination willingness were analyzed using descriptive statistics. The pandemic follow-up data on disease activity, as well as mental, physical, and social burden, were analyzed in the same manner. Chi-square and Fisher’s exact tests were used to test for significant differences between patients with and without probable PTSD in categorical variables. These variables included the number of comorbidities, the presence of psychiatric comorbidities, sex, employment status, smoking history, disease course, presence of polypharmacy, DMT use, willingness regarding recommended standard vaccinations, pre-survey MS activity, and mental/physical/social burden. Additionally, Mann–Whitney *U* tests were used to compare age, schooling, disease duration, EDSS score, total number of medications used, NEO-FFI scores, TCI-R scores, and HADS scores between patients with and without probable PTSD. We conducted a robustness analysis by separately examining women and men to check whether differences in psychometric measures are consistent across both sexes. Stuart-Maxwell tests were used to compare the responses in the adapted TSQ between the first and second follow-up, and generalized linear mixed-effects models with logistic link function were used to assess whether a given predictor is associated with the change in the TSQ-based classification over time. Due to the small number of CIS cases, we combined patients with relapsing-remitting MS (RRMS) and patients with CIS into a single group of relapsing MS for the statistical analysis. The significance level was set at α = 0.05.

## 3. Results

### 3.1. Prevalence of Probable PTSD During the SARS-CoV-2 Pandemic in Patients with MS

The first follow-up survey was conducted three months after the WHO declared the COVID-19 outbreak a global pandemic. Two hundred pwMS participated in the first follow-up survey. The second follow-up survey was conducted approximately one year after the approval of the first SARS-CoV-2 vaccines, with 149 out of the 200 pwMS participating again. A total of 51 pwMS were lost to follow-up. In the adapted TSQ survey at both the first and second follow-up, fewer than 10 patients responded with “very strongly” to any individual question (Supplementary Table S1). At the second follow-up, the patients more often responded with “(very) strongly” to the question about feeling jumpy or easily startled by something unexpected (Stuart-Maxwell test *p* < 0.001). On the other hand, a significantly higher proportion of patients answered “not at all” to the questions about sleep difficulties, concentration problems, and heightened awareness of danger at the second follow-up (*p* < 0.05). Supplementary Table S2 shows the frequency of dichotomized responses for each item of the adapted TSQ at the two follow-up time points, along with the range of scores of the adapted TSQ and the prevalence of probable PTSD at a cut-off score of 6 or higher.

**Figure 1:**
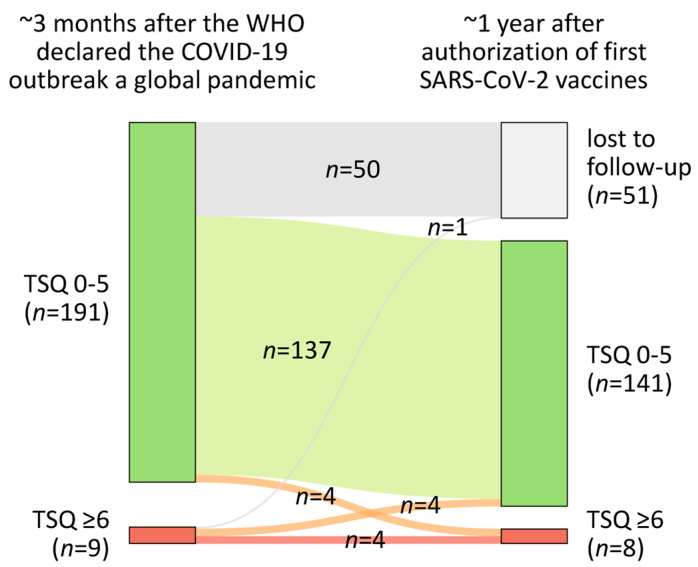
Occurrence of probable PTSD in MS patients during the SARS-CoV-2 pandemic. The size of the colored boxes corresponds to the number of pwMS with and without probable PTSD in both follow-ups. A total of 200 pwMS were interviewed approximately 3 months after the WHO declared the COVID-19 outbreak a global pandemic (first follow-up). In the second follow-up, conducted approximately 1 year after the authorization of the first SARS-CoV-2 vaccines, 149 pwMS were interviewed again, while 51 pwMS were lost to follow-up. Out of the 149 patients who participated in both surveys, 12 were classified as having probable PTSD (i.e., they had a score of at least 6 on the adapted version of the TSQ). COVID-19, coronavirus disease 2019; MS, multiple sclerosis; PTSD, post-traumatic stress disorder; pwMS, people with MS; SARS-CoV-2, severe acute respiratory syndrome coronavirus type 2; TSQ, adapted Trauma Screening Questionnaire; WHO, World Health Organization.

Probable PTSD was identified in 9 out of 200 pwMS (4.5%) at the first follow-up and in 8 out of 149 pwMS (5.4%) at the second follow-up. Among these, four patients had probable PTSD at both follow-ups, resulting in a total of 13 patients with probable PTSD. Four pwMS transitioned from probable PTSD at the first follow-up to no probable PTSD at the second follow-up, four changed from no probable PTSD to probable PTSD, and one was lost to follow-up (Figure 1).

### 3.2. Demographic and Clinical Comparison of MS Patients with and Without Probable PTSD

The study included 149 participants with pwMS who participated in both follow-ups (*n* = 149), 64.4% of whom were female, with a median age at baseline of 51 years (Table 1). The median disease duration was 10 years, and 69.1% of the patients had been diagnosed with relapsing MS. The median EDSS score at baseline was 3.5. More than half of the participants (51.7%) reported polypharmacy (use of at least 5 drugs), and 78.5% were receiving a DMT for the treatment of MS (most used was interferon beta: 12.8%). At baseline, 75.2% of the pwMS were willing to comply with recommended standard vaccinations. In the 3 months prior to the follow-up surveys, 12.8% reported experiencing relapses (prior to one follow-up: *n* = 18, prior to both follow-ups: *n* = 1), and 32.7% experienced disease progression (prior to one follow-up: *n* = 34, prior to both follow-ups: *n* = 14).

Out of the 149 pwMS who participated in both follow-up surveys, 12 (8.1%) were classified as having probable PTSD in at least one follow-up survey. A significantly higher proportion of patients with probable PTSD had ever smoked (100.0% vs. 48.6%, Fisher’s exact test *p* = 0.027) and had psychiatric comorbidities (66.7% vs. 12.4%, *p* < 0.001), especially depression (58.3% vs. 11.7%, *p* < 0.001), than those without probable PTSD (Table 1). However, ever-smoking and the presence of psychiatric comorbidities were not significantly associated with the change in the status of probable PTSD over time (*p* > 0.4 in generalized linear mixed-effects models). The following differences between pwMS stratified by probable PTSD were also not statistically significant: Patients with probable PTSD were more frequently female (75.0% vs. 63.5%) and had a higher median EDSS score (3.75 vs. 3.5) as well as a higher median number of medications used (8 vs. 5) than patients without probable PTSD. Furthermore, patients with probable PTSD presented higher proportions of relapsing MS (83.3% vs. 67.9%), multimorbidity (presence of at least two comorbidities: 75.0% vs. 53.3%), polypharmacy (66.7% vs. 50.4%) and MS activity within the last 3 months (relapse: 25.0% vs. 11.7%; progression: 58.3% vs. 30.4%). Patients without probable PTSD were older (median: 48 vs. 51 years), more often employed (50.4% vs. 41.7%), and more frequently willing to comply with standard vaccination recommendations (76.6% vs. 58.3%). They also showed a higher prevalence of DMT use (79.6% vs. 66.7%; also, no significant differences with regard to the single DMTs), while both patient groups showed the same median disease duration of 10 years and median schooling period of 10 years.

### 3.3. Probable PTSD in Association with Personality Characteristics, Symptoms of Anxiety and Depression and Mental, Physical, and Social Burden

**Figure 2:**
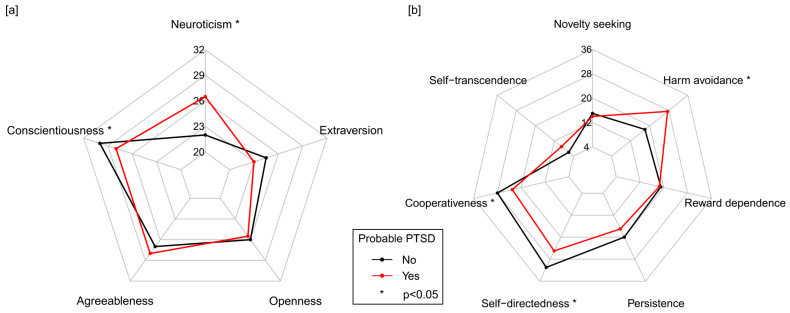
Personality characterization of MS patients with and without probable PTSD using the following: (**a**) NEO-FFI; (**b**) TCI-R. A total of 149 participants were split into patients with probable PTSD (*n* = 12) and those without probable PTSD (*n* = 137). These spider plots visualize the median values of (**a**) NEO-FFI and (**b**) TCI-R dimension scores per patient subgroup, with the minimum values of the variables being displayed in the center and the maximum values at the outer rim. Missing values in the dimensions of NEO-FFI (*n* = 3) and TCI-R (up to *n* = 16) were omitted from the analysis. Patients with probable PTSD had significantly higher median scores in neuroticism (NEO-FFI; 26.5 vs. 22, *p* = 0.006) and harm avoidance (TCI-R; 27.5 vs. 18, *p* = 0.008), while in patients without probable PTSD, median scores in conscientiousness (NEO-FFI; 28 vs. 30, *p* = 0.030), cooperativeness (TCI-R; 23 vs. 28, *p* = 0.008) and self-directedness (TCI-R; 25 vs. 31, *p* = 0.022) were significantly higher. MS, multiple sclerosis; NEO-FFI, NEO-Five Factor Inventory; *p*, *p*-value calculated using the Mann–Whitney *U* test; PTSD, post-traumatic stress disorder; TCI-R, Temperament and Character Inventory-Revised; *, *p* < 0.05.

In pwMS who participated in both follow-ups, the highest median score (range) of the NEO-FFI dimensions at baseline occurred for conscientiousness (30 (13–45)), followed by agreeableness (27 (14–42)), openness (26 (12–45)), extraversion (24 (5–41)) and neuroticism (22 (9–46)). Among the TCI-R dimensions, the highest score at baseline occurred for self-directedness (30.5 (8–40)), followed by cooperativeness (28 (12–35)), persistence (19 (0–31)), harm avoidance (19 (4–32)), reward dependence (19 (4–27)), novelty seeking (15 (0–28)) and self-transcendence (6 (1–20)) (Supplementary Table S3). In patients with probable PTSD, significantly higher median scores of neuroticism (26.5 vs. 22, Mann–Whitney *U* test: *p* = 0.006) and harm avoidance (27.5 vs. 18, *p* = 0.008) and significantly lower scores of conscientiousness (28 vs. 30, *p* = 0.030), cooperativeness (23 vs. 28, *p* = 0.008) and self-directedness (25 vs. 31, *p* = 0.022) were found compared to pwMS without probable PTSD (Figure 2). When considering women, men, and patients with and without psychiatric comorbidity, the same personality dimensions differed significantly only between female patients with and without probable PTSD (Supplementary Table S4, Supplementary Figure S1). Overall, similar differences were observed in male pwMS, patients with psychiatric comorbidity, and those without psychiatric comorbidity. However, due to the smaller sample size, these disparities did not attain statistical significance. For agreeableness (28 vs. 27, *p* = 0.177), extraversion (23 vs. 24.5, *p* = 0.179), openness (25.5 vs. 26, *p* = 0.705), novelty seeking (14 vs. 15, *p* = 0.676), persistence (17 vs. 20, *p* = 0.166), reward dependence (18.5 vs. 19, *p* = 0.223) and self-transcendence (9 vs. 6, *p* = 0.286), the differences between patients with and without probable PTSD were not statistically significant in the overall study population. Detailed summary statistics of the NEO-FFI and TCI-R scores are provided in Supplementary Tables S3 and S4, and the distribution of personality trait data is visualized in Supplementary Figure S2.

**Figure 3:**
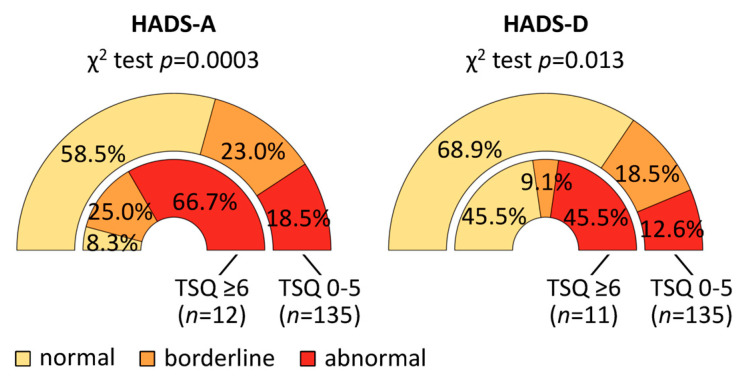
Anxiety and depression levels in MS patients with and without probable PTSD. A total of 149 pwMS completed the modified TSQ, a brief screening tool used to detect PTSD, both early and later in the SARS-CoV-2 pandemic. Those with a TSQ score of 6 or higher were classified as having probable PTSD. Additionally, the majority of these 149 pwMS completed the HADS at baseline, i.e., prior to the start of the SARS-CoV-2 pandemic. The subscale sum scores of the HADS were categorized as normal (0–7 points), borderline (8–10 points) or abnormal (11–21 points). Patients with probable PTSD had significantly more often abnormal anxiety (HADS-A) and depression (HADS-D) scores than those without probable PTSD. Only valid data are shown in the graph, omitting patients with missing values for HADS-A (*n* = 2) or HADS-D (*n* = 3). HADS, Hospital Anxiety and Depression Scale; MS, multiple sclerosis; *n*, number of patients; *p*, *p*-value; pwMS, people with MS; PTSD, post-traumatic stress disorder; SARS-CoV-2, severe acute respiratory syndrome coronavirus type 2; TSQ, adapted Trauma Screening Questionnaire.

The median values of the HADS scores were found to be significantly higher in patients with probable PTSD than in those without probable PTSD (HADS-A: 11.5 vs. 7, Mann–Whitney *U* test *p* = 0.001; HADS-D: 8 vs. 5, *p* = 0.011) (Supplementary Table S3). More precisely, patients with probable PTSD showed significantly higher rates of abnormal HADS-A (66.7% vs. 18.5%) and HADS-D scores (45.5% vs. 12.6%) compared to those without probable PTSD, who had higher rates of normal HADS-A (58.5% vs. 8.3%) and HADS-D scores (68.9% vs. 45.5%) (chi-square test: HADS-A *p* = 0.0003, HADS-D *p* = 0.013) (Figure 3). The differences in HADS scores were more pronounced among the women (Supplementary Table S4, Supplementary Figure S1).

PwMS reported high levels of mental, physical, and social burden during the SARS-CoV-2 pandemic, with 20–30% being affected (Table 2). Patients with probable PTSD reported severe or very severe mental, physical, and social burden significantly more often than patients without probable PTSD at both follow-ups (Fisher’s exact tests *p* ≤ 0.045). Among patients with probable PTSD, the reported mental and physical burden remained unchanged proportionally over the course of the SARS-CoV-2 pandemic, while the social burden increased slightly (from 7 to 8 pwMS) over the course of the follow-up surveys. In patients without probable PTSD, the proportion of perceived social burden decreased from the first to the second follow-up.

## 4. Discussion

The SARS-CoV-2 pandemic has significantly impacted health, politics, and society [15]. It has resulted in increased burdens for people worldwide, including social restrictions implemented by governments to address public health concerns [72]. We examined the traumatizing impact of the pandemic on individuals with a chronic illness, specifically pwMS. The occurrence of probable PTSD, indicated by scores of 6 or higher on an adapted version of the TSQ, was assessed at two different times or situations during the pandemic: approximately 3 months after the WHO declared COVID-19 a pandemic (i.e., before the availability of indication-specific vaccinations) and 1 year after the availability of the first approved SARS-CoV-2 vaccinations in Germany.

Trauma can lead to illness in the form of PTSD. However, not everyone who experiences trauma will develop PTSD [64,65]. Common risk factors for PTSD include social discrimination, poor health [12], living alone, and the risk of reduced economic stability [73,74,75]. Loneliness is a public health problem that is associated with poor physical and mental health and even extreme levels of it [8]. These findings are consistent with a study that identified psychological factors as precursors of poor mental health [76]. Protective factors include social support, being married, satisfaction with health information, and the effectiveness of government interventions to combat infections [70,77,78].

The lifetime prevalence of PTSD in the general population ranges from 5–6% in men to 10–12% in women [79,80]. However, rates vary widely among epidemiological studies, ranging from less than 1% in Nigeria [74] or Switzerland [75] to approximately 5–9% in the United States [81], the Netherlands [82] and Norway [76] and as high as 37% in post-conflict countries such as Ethiopia, Gaza, Algeria and Cambodia [83]. A review of studies examining the SARS-CoV-2 pandemic as a traumatic event found a wide range of PTSD rates in the general population, ranging from 10.8% to 67.1% [84]. The reported rate of PTSD during the SARS-CoV-2 pandemic was higher than in previous epidemics [84], such as SARS-CoV-1 in 2000 (25.5%) [85], MERS in 2019 (26.9–42.3%) [86] and Ebola in 2020 (21%) [87]. Studies based on the TSQ reported trauma-related symptoms (TSQ score ≥ 6) in up to 37.2% of confirmed COVID-19 cases [69,88] and up to 23.3% of healthcare workers [89,90] during the SARS-CoV-2 pandemic. A higher prevalence of probable PTSD (TSQ score ≥ 6) was observed in individuals living with frailty one year after COVID-19 hospital admission [91] as well as in those who did not receive a SARS-CoV-2 vaccination compared to vaccinated individuals (15.3% vs. 12.6%) [92]. We are not aware of a previous study that specifically utilized the TSQ in the context of MS. However, in our study, the proportion of pwMS with probable PTSD over the course of the pandemic (8.1%) was much lower compared to the rates reported in the aforementioned studies. It is surprising that pwMS were not found to be at a greater risk of contracting SARS-CoV-2 than the general population [93] despite their compromised immune system associated with the autoimmune disease [34]. The prevalence of PTSD varies significantly depending on the specific chronic disease. For instance, the proportion of probable PTSD in MS in our study was roughly comparable to that in cardiovascular diseases (6.6%) but was far lower than that in cerebrovascular diseases (23.6%) [94]. In individuals experiencing chronic pain, the pooled prevalence of PTSD averages at 9.7%, with the potential to reach 20.5% depending on the specific type of pain [95,96]. A similar trend is observed in epilepsy, where the pooled PTSD prevalence is 18%, though this value is also subject to variation depending on the specific type of epilepsy [97]. The relatively low rate of probable PTSD observed in the present study is also apparent in two studies investigating the prevalence of PTSD in pwMS. Ostacoli et al. examined 232 pwMS, 5.17% of whom were diagnosed with PTSD [45]. The analysis of 988 pwMS by Carletto et al. yielded notable findings: 25.5% of pwMS reported posttraumatic symptoms, while in 5.7% to 8.5% of the study population, the PTSD diagnosis was confirmed [46]. This substantial discrepancy between reported symptoms and confirmed diagnosis suggests the possibility that the proportion of pwMS with probable PTSD, as determined by our study, may also be lower after an additional diagnostic process, i.e., the proportion of probable PTSD in our study may be somewhat overestimated. The variation in reported PTSD rates could be attributed to the utilization of different diagnostic instruments in the aforementioned studies. Previous studies, which focused on epidemics and trauma assessment, utilized the Impact of Event Scale-Revised, the PTSD module of the Structured Clinical Interview for Diagnostic and Statistical Manual of Mental Disorders (4th edition, text revision), the Clinician-Administered PTSD Scale and the PTSD Checklist [45,46,85,86,87]. In addition, the studies of non-MS patients were mostly conducted on survivors of the respective epidemics. However, this was not a prerequisite for participating in our survey. It may be speculated that the pwMS included in our study may not have been fully cognizant of the issue to the degree that it could have resulted in PTSD in relation to the SARS-CoV-2 pandemic.

Depression is a common psychiatric condition in pwMS, with a lifetime risk of 27.01%, according to a meta-analysis by Peres et al. [98]. The lifetime prevalence of major depression in pwMS is thus considerably higher than that of the general U.S. population, which ranges from 10.4% to 20.6% [99]. According to a Canadian population-based study, age- and sex-adjusted prevalence rates of major depression were twice as high in pwMS as in patients with other chronic diseases [100]. Compared to patients with other chronic neurological diseases, pwMS also exhibit more severe depressive symptoms [101,102]. PTSD has been associated with depression [103]. Individuals who have experienced trauma are more susceptible to developing depression and vice versa [12,104,105]. The SARS-CoV-2 pandemic has made it challenging to maintain social relationships due to restrictions on physical contact with family and friends as well as limitations on social and leisure activities [106]. It is important to note that these challenges were not limited to a specific group of people. The international evidence suggests that the SARS-CoV-2 pandemic has negatively impacted global mental health. Research suggests that depression, anxiety, and PTSD can negatively affect the mental well-being of the general population [14]. Our study found that pwMS with probable PTSD showed abnormal scores in HADS-D more often (45.5%) compared to those without probable PTSD (12.6%). Factors that contribute to the association between MS and depressive disorders include the psychosocial impact of disability due to MS [107], the direct effects of lesions on brain structures involved in mood regulation and maintenance [11,108], and the potential mood fluctuations associated with some DMTs like interferon beta used in MS treatment [109,110]. Additionally, dysfunction of the immune system may also play a role. Furthermore, feelings of helplessness and dependence on others may contribute to a higher prevalence of depressive disorders [111]. This is compounded by the inclination toward isolation resulting from the restrictions on contact imposed by the government during the SARS-CoV-2 pandemic [112,113,114].

The lifetime prevalence of anxiety disorders in the general population ranges from 20% to 30% [115,116]. Several studies have reported a positive correlation between the co-occurrence of trauma and anxiety disorders [12,104,105,117,118,119,120]. Anxiety disorders often co-occur with other mental disorders, particularly depression [121]. According to a comprehensive systematic review [122], anxiety disorder is the second most prevalent comorbidity in MS, affecting 21.9%. Our study revealed a significant difference in the prevalence of abnormal HADS-A scores between individuals with probable PTSD at 66.7% and those without probable PTSD at 18.5%. In comparison, in a study of people affected by Hurricane Katrina, all respondents who were classified as having PTSD using the TSQ (5-point scale) were also classified as having an anxiety-mood disorder [67]. Pre-existing affective conditions such as anxiety or depression are reported to be associated with an increased risk of PTSD [123,124,125], as these individuals may exhibit a heightened sensitivity to psychological stress. In the present study, patients with probable PTSD demonstrated a fivefold higher proportion of pre-pandemic psychiatric comorbidities compared to those without probable PTSD (66.7% vs. 12.4%). Shared genetic vulnerabilities could also provide an explanation for the link between PTSD and affective disorders [126]. Clinically, individuals afflicted with both PTSD and affective disorders frequently exhibit more pronounced symptoms, functional limitations, and a diminished quality of life [126]. These findings underscore the necessity of a comprehensive treatment approach that considers both PTSD and concomitant disorders. A plethora of studies have demonstrated the efficacy of psychotherapeutic methods, such as exposure therapy and cognitive behavioral therapy, particularly when administered within the initial three months following a traumatic incident [127]. Furthermore, multidisciplinary approaches have demonstrated efficacy in alleviating symptoms associated with PTSD and depression. Telemedical forms of treatment also represent a promising alternative to conventional therapy, especially for individuals with limited access to face-to-face sessions. A substantial body of research has demonstrated that the efficacy of telemedicine interventions is comparable to traditional face-to-face therapies [128]. However, beyond individual psychological factors, external influences—such as societal and environmental stressors—also play a crucial role in shaping mental health outcomes. The context in which our study was conducted, particularly the SARS-CoV-2 pandemic, may have contributed to the observed increase in anxiety disorders in the general population [106]. A study conducted in the UK with a sample size of 34,465 individuals reported that approximately 55% of the participants experienced worsening depression and anxiety symptoms from the period before to the time during the SARS-CoV-2 pandemic [129]. Having a previous mental health diagnosis and taking psychiatric medication were found to be associated with “maladaptive” trajectories of stress, anxiety, and depression during the SARS-CoV-2 pandemic [130]. The pandemic posed a significant health risk for pwMS due to their dysfunctional immune system resulting from the autoimmune disease [31]. Additionally, DMTs may increase the susceptibility to infections [25,39,42,131]. To prevent exacerbating fear and inappropriate behavior in vulnerable populations, it is crucial to limit the repetition of frightening information on infectious diseases in the media. Strategies should be explored to manage anxiety-inducing media consumption and promote adherence to health advice while minimizing anxiety. The use of digital interventions for sleep or stress management could also be taken into consideration. It should be noted that mechanisms that modify anxiety operate at different levels, including molecular, neurobiological, cognitive, behavioral, and social levels [106,132].

We also found that perceived mental, physical and social burden were particularly high among pwMS with probable PTSD. Stress associated with trauma can lead to changes in the brain. These changes include a smaller volume of the hippocampus. The hippocampus is sensitive to stress and plays a key role in declarative memory [108,133,134]. Given that pwMS often report increased fatigue associated with autoimmune-mediated CNS disease [135,136,137] and are also more prone to dementia, co-existing PTSD exacerbates these symptoms.

When analyzing the personality traits of the NEO-FFI dimensions in our study, we could observe that pwMS with possible PTSD had significantly higher scores for neuroticism compared to pwMS without possible PTSD. A high neuroticism score on the NEO-FFI is associated with an increased risk of developing PTSD and enhanced pandemic-related stress responses [138]. Neuroticism is a personality dimension that is characterized by emotional instability, susceptibility to stress, negative affect, anxiety, and depression [139,140,141]. Those with a higher neuroticism score tend to experience more intense emotional reactions. Consequently, they experience more intense negative emotions, such as anxiety, sadness, and anger. Such intensified emotional reactions can impede the processing of traumatic experiences. Additionally, difficulties with emotion regulation are observed. Those with these difficulties may be less able to cope effectively, which can result in an exacerbation of PTSD symptoms. Negative cognitive styles are among the characteristics that define the construct of neuroticism. These can perpetuate or intensify PTSD symptoms by fostering a pervasive negative self-perception and self-efficacy. In summary, those with high neuroticism are more susceptible to developing PTSD, and they are generally more sensitive to stress and emotional stress.

A high score on the harm avoidance subscale of the TCI-R may have several important implications for individuals at increased risk for PTSD. Harm avoidance is a temperamental dimension that is characterized by a tendency to avoid situations that may result in punishment, danger, or unpleasant experiences. Anticipatory worry and pessimism, fear of uncertainty, shyness with strangers, as well as fatigability and asthenia are the four lower-order traits of harm avoidance [55]. Heightened sensitivity can result in individuals experiencing traumatic events with greater intensity and reacting more strongly to them. Such individuals may exhibit avoidance behavior. This is more prevalent after a traumatic event, impeding the ability to confront and process the trauma. Individuals with high scores on this dimension thus frequently encounter difficulties in coping with stress. This can impair their ability to effectively address the consequences of a traumatic event. In conclusion, a high harm avoidance score on the TCI-R may indicate an increased susceptibility to PTSD and underscores the necessity for targeted preventive and therapeutic interventions to promote emotional stability and the capacity to cope with stress.

Our study also revealed significantly lower scores for the TCI-R dimensions of cooperativeness and self-directedness in pwMS with possible PTSD. Individuals with these personality characteristics tend to socially distance themselves, reinforcing existing difficulties in being open with others. Traumatic experiences can lead to an increased mistrust of others, making it difficult for individuals to form healthy relationships and seek support when needed. This can also manifest in difficulties when working with others, often due to a lack of empathy toward others [142]. Consequently, those affected should be provided with the necessary support to develop and utilize social support systems. Social support can serve to diminish feelings of isolation and facilitate coping strategies. Furthermore, it is crucial for these individuals to learn techniques that enhance mindfulness and self-care, as this will enhance their overall stress resilience and improve their ability to cope with negative emotions. Techniques such as meditation, yoga, and regular exercise have been demonstrated to be beneficial in this regard [143].

MS-related brain changes may increase susceptibility to PTSD. These changes may impair neurological function and affect stress processing. Whether the expression of certain personality traits, such as neuroticism, harm avoidance, and conscientiousness, could be a consequence of this should be discussed in the following. PwMS with PTSD show higher disease activity (MS relapses and new lesions), and disability progression is faster in these patients, which may indicate neurological distress [144]. Relapses are also a risk factor for the development of severe PTSD in newly diagnosed pwMS [145]. Studies by Ogle et al. show that neuroticism may trigger PTSD symptoms through increased emotionality, availability, and centrality of trauma memories, as described in mnemonic PTSD models [146]. In addition, higher neuroticism scores have been shown to be associated with increased activity in certain brain regions in women with PTSD following abuse, particularly in the processing of negative emotions [147]. However, these studies of neuroticism and PTSD are not related to MS. High harm avoidance scores may negatively influence stress processing and be associated with increased susceptibility to PTSD in pwMS. These patients may react more strongly to stress due to their avoidance tendencies, further increasing neurological distress. Low conscientiousness is associated with cognitive dysfunction and neuropsychiatric symptoms in MS and may, therefore, influence the relationship between brain gray matter and symptoms such as euphoria [148]. In addition, low scores on conscientiousness may lead to more rapid cognitive decline, while higher scores may slow cognitive decline [149]. The combination of MS-related brain changes and low conscientiousness may increase susceptibility to PTSD by increasing cognitive and emotional distress. This may make psychological adjustment to the disease more difficult and impair the quality of life [150].

Additional confounding factors, such as previous trauma and socioeconomic status, could influence the results of our study. In the event that people have previously experienced trauma, there is a possibility that they may exhibit a higher prevalence of PTSD [151,152,153,154], as they may have developed a heightened sensitivity to traumatic events. A lower socioeconomic status may be associated with a higher prevalence of PTSD [155,156,157], as people with a lower socioeconomic status may have less access to psychological support and treatment, as well as a reduced availability of protective resources such as social support. It is essential to note that these confounders are only hypotheses, and we do not have data to confirm them in pwMS. However, it would be crucial to consider these factors in future studies to gain a more accurate understanding of the prevalence of PTSD among pwMS.

The study has some limitations, including a limited sample size. The validity of the data may be limited, as most of it was collected through patient self-reports. The bicentric study design may have caused a documentation bias during the structured interview among the centers and may have limited the direct transferability of the findings to broader MS populations, particularly those in different healthcare systems or sociocultural contexts. Patient care varies across countries; for example, Germany and France have a well-established inpatient healthcare system, while treatment in the United Kingdom and Canada is primarily provided in outpatient settings [158,159]. Depending on the country, government agencies and health insurance providers do not fully cover the costs of therapies, and treatment access is largely influenced by their costs. Socioeconomic status and cultural background can influence the perception of and response to trauma as well as coping with psychological stress. In addition, only patients who were inpatients or outpatients at one of the two MS centers were included. Thus, it is not possible to make any conclusions about the trauma experiences of patients seen by neurologists in private practice. Future research incorporating multinational cohorts would be valuable to further explore the generalizability of our results across diverse healthcare settings and populations. Furthermore, the adaptation of the TSQ with regard to the SARS-CoV-2 pandemic was not validated. The adaptation of the TSQ (i.e., the rewording of the questions related to the SARS-CoV-2 pandemic, the use of an undefined time period for the questions, the implementation of a Likert scale for the answer options, and the data binarization) inherently impacts the estimated prevalence of probable PTSD. On the one hand, the methodological adaptation of the TSQ with the reclassification of the 5-point scale into a 2-point scale could lead to a lower sensitivity to detect probable PTSD. Selecting only the responses “strongly” or “very strongly” as indicators of probable PTSD while excluding the “not sure” category is a strategy used to identify cases with greater certainty, but there is a risk of losing some cases of probable PTSD. Moreover, the implementation of further trauma-oriented diagnostics, including the distinction between post-traumatic symptoms and confirmed PTSD, may be likely to result in a lower prevalence of actual PTSD. Given the onset of the pandemic during the baseline survey period, we proactively initiated the assessment of pandemic-related traumatic burden. Our approach was driven by the absence of a pandemic-specific instrument to assess such a burden. It is imperative to reiterate that the utilization of the adapted TSQ is intended for the monitoring of trends and does not constitute a clinical diagnostic criterion of PTSD. The statistical power of comparing the two subgroups of patients with and without probable PTSD is limited due to the unequal size of the subgroups. To enhance the statistical validity of the findings, future studies should consider linking the analysis to national or international MS registries. Additionally, to facilitate comparisons with other studies that performed epidemic surveys, future studies may also use the Impact of Event Scale—Revised [160,161] and the PTSD Checklist for DSM-5 [162], which are other questionnaires commonly used to assess trauma. This would help broaden the recording of trauma criteria and strengthen conclusions regarding the presence or absence of PTSD in pwMS. However, our study is a significant advancement in researching the impact of the pandemic on mental health, especially the trauma burden on pwMS during the SARS-CoV-2 pandemic. To our knowledge, it is the first study that longitudinally evaluated traumatic stress at two points in time during the pandemic in patients with MS, and we hope that our findings will inform the development of more robust and validated instruments for assessing pandemic-related traumatic burden in the future.

## 5. Conclusions

This study was the first to analyze the occurrence of probable PTSD in pwMS over time during the SARS-CoV-2 pandemic. We identified significant differences in the personality traits of pwMS depending on their pandemic-associated trauma status. Our findings further indicated extraordinarily high levels of pre-pandemic symptoms of anxiety and depression among pwMS with probable PTSD. Furthermore, pwMS with probable PTSD experienced significantly increased mental, physical, and social burdens compared to those without probable PTSD. Our study has implications for the evaluation of support needs on social, psychological, and physical levels. To promote the well-being of pwMS, multidisciplinary teams need to improve their support for those affected by the pandemic and who are more vulnerable to the negative effects of social stress. Due to the importance of vaccination in maintaining health, particularly for pwMS [163,164], it is crucial to intensify the dissemination of information to patients with comorbid PTSD. Additionally, psycho- and socio-therapeutic services should be provided. Further research, particularly longitudinal studies, is necessary to investigate the impact of trauma and probable PTSD on mental health and self-care, such as vaccination advice, to improve the quality of life for individuals with MS.

## Figures and Tables

**Table 1:** Patient characteristics and comparison of MS patients with and without probable PTSD.

Characteristic	Total (*n* = 149)	Probable PTSD (*n* = 12)	No Probable PTSD (*n* = 137)	*p*
Sex, *n* (%)				0.540 ^Fi^
Men	53 (35.6)	3 (25.0)	50 (36.5)	
Women	96 (64.4)	9 (75.0)	87 (63.5)	
Age (years), median (range)	51 (20–74)	48 (29–57)	51 (20–74)	0.184 ^U^
School years, median (range)	10 (8–14)	10 (10–14)	10 (8–13)	0.875 ^U^
Employment, *n* (%)	74 (49.7)	5 (41.7)	69 (50.4)	0.765 ^Fi^
EDSS score, median (range)	3.5 (0.0–8.5)	3.75 (1.0–6.5)	3.5 (0.0–8.5)	0.578 ^U^
Disease duration (years), median (range)	10 (0–37)	10 (1–31)	10 (0–37)	0.756 ^U^
Disease course, *n* (%)				0.344 ^Fi^
Relapsing MS	103 (69.1)	10 (83.3)	93 (67.9)	
Progressive MS	46 (30.9)	2 (16.7)	44 (32.1)	
Number of comorbidities, *n* (%)				0.340 ^Chi^
0	39 (26.2)	2 (16.7)	37 (27.0)	
1	28 (18.8)	1 (8.3)	27 (19.7)	
≥2	82 (55.0)	9 (75.0)	73 (53.3)	
Psychiatric comorbidities, *n* (%)	25 (16.8)	8 (66.7)	17 (12.4)	**<0.001** **^Fi^**
Anxiety disorder *^1^	5 (3.4)	2 (16.7)	3 (2.2)	0.052 ^Fi^
Depression *^1^	23 (15.4)	7 (58.3)	16 (11.7)	**<0.001** **^Fi^**
Drugs in total, median (range)	5 (0–16)	8 (2–12)	5 (0–16)	0.113 ^U^
Polypharmacy (use of ≥5 drugs), *n* (%)	77 (51.7)	8 (66.7)	69 (50.4)	0.371 ^Fi^
Use of DMT, *n* (%)	117 (78.5)	8 (66.7)	109 (79.6)	0.288 ^Fi^
Alemtuzumab	1 (0.7)	0 (0.0)	1 (0.7)	1.000 ^Fi^
Azathioprine	1 (0.7)	0 (0.0)	1 (0.7)	1.000 ^Fi^
Cladribine	2 (1.3)	0 (0.0)	2 (1.5)	1.000 ^Fi^
Dimethyl fumarate	10 (6.7)	2 (16.7)	8 (5.8)	0.132 ^Fi^
Fingolimod	13 (8.7)	1 (8.3)	12 (8.8)	1.000 ^Fi^
Glatiramer actetate	13 (8.7)	1 (8.3)	12 (8.8)	1.000 ^Fi^
Glucocorticosteroid pulse therapy	16 (10.7)	0 (0.0)	16 (11.7)	0.364 ^Fi^
Interferon beta	19 (12.8)	1 (8.3)	18 (13.1)	1.000 ^Fi^
Intravenous immunoglobulin	1 (0.7)	0 (0.0)	1 (0.7)	1.000 ^Fi^
Mitoxantrone	3 (2.0)	0 (0.0)	3 (2.2)	1.000 ^Fi^
Natalizumab	15 (10.1)	1 (8.3)	14 (10.2)	1.000 ^Fi^
Ocrelizumab	11 (7.4)	1 (8.3)	10 (7.3)	1.000 ^Fi^
Teriflunomide	12 (8.1)	1 (8.3)	11 (8.0)	1.000 ^Fi^
Ever smoked, *n* (%) *^2^	57 (51.4)	6 (100.0)	51 (48.6)	**0.027** **^Fi^**
Willingness regarding recommended standard vaccinations, *n* (%)	112 (75.2)	7 (58.3)	105 (76.6)	0.173 ^Fi^
MS relapses within the last 3 months prior to the surveys	19 (12.8)	3 (25.0)	16 (11.7)	0.183 ^Fi^
MS progression within the last 3 months prior to the surveys *^3^	48 (32.7)	7 (58.3)	41 (30.4)	0.059 ^Fi^

^Chi^, chi-square test; DMT, disease-modifying therapy; EDSS, Expanded Disability Status Scale; ^Fi^, Fisher’s exact test; MS, multiple sclerosis; *n*, number of patients; *p*, *p*-value; PTSD, post-traumatic stress disorder; ^U^, Mann–Whitney *U* test; *^1^, 3 patients had both anxiety disorder and depression as psychiatric comorbidity; *^2^, denominators vary due to missing values (total: *n* = 38, probable PTSD: *n* = 6, no probable PTSD: *n* = 32); *^3^, denominators vary due to missing values (total: *n* = 2, no probable PTSD: *n* = 2).

**Table 2:** Frequency of mental, physical, and social burden shortly prior to the surveys among MS patients stratified by probable PTSD status, *n* (%).

Burden	Total (*n* = 149)	Probable PTSD (*n* = 12)	No Probable PTSD (*n* = 137)	*p* ^Fi^
^~^3 months after the COVID-19 outbreak was declared a global pandemic				
Severe or very severe mental burden	44 (29.5)	10 (83.3)	34 (24.8)	**<0.001**
Severe or very severe physical burden	42 (28.2)	8 (66.7)	34 (24.8)	**0.004**
Severe or very severe social burden	45 (30.2)	7 (58.3)	38 (27.7)	**0.045**
^~^1 year after the authorization of first SARS-CoV-2 vaccines				
Severe or very severe mental burden	38 (25.5)	10 (83.3)	28 (20.4)	**<0.001**
Severe or very severe physical burden	44 (29.5)	8 (66.7)	36 (26.3)	**0.006**
Severe or very severe social burden	35 (23.5)	8 (66.7)	27 (19.7)	**0.001**

COVID-19, coronavirus disease 2019; ^Fi^, Fisher’s exact test; *n*, number of patients; PTSD, post-traumatic stress disorder; SARS-CoV-2, severe acute respiratory syndrome coronavirus type 2.

## Data Availability

The datasets generated and analyzed in the current study are available from the corresponding author on reasonable request.

## Supplementary Materials

The following supporting information can be downloaded at https://www.mdpi.com/article/10.3390/jcm14082665/s1, Figure S1: Verification of differences in personality traits and HADS scores in subgroups; Figure S2: Distribution of personality trait data from the [a] NEO-FFI and [b] TCI R questionnaires in patients with MS; Table S1: Frequency of responses per item of the adapted TSQ (5-point scale) during the SARS-CoV-2 pandemic among MS patients; Table S2: Frequency of responses after dichotomization per item of the adapted TSQ (2-point scale) and the distribution of TSQ scores during the SARS-CoV-2 pandemic among MS patients; Table S3: Comparison of MS patients with and without probable PTSD regarding NEO-FFI and TCI-R dimensions as well as HADS scores; Table S4: Comparison of MS patients with and without probable PTSD regarding NEO-FFI and TCI-R dimensions as well as HADS scores, stratified by sex.
