# Supplementary material for: Trauma Burden Affected People with Multiple Sclerosis During SARS-CoV-2 Pandemic
Source: J Clin Med. 2025 Apr 13;14(8):2665. doi: 10.3390/jcm14082665 (PMC12027752; doi:10.3390/jcm14082665)
Supplement: Supplementary file 1 [file jcm-14-02665-s001.zip › Supplementary_Figure_S1.pdf]

Supplementary Figure S1. Verification of differences in personality traits and HADS scores in subgroups.

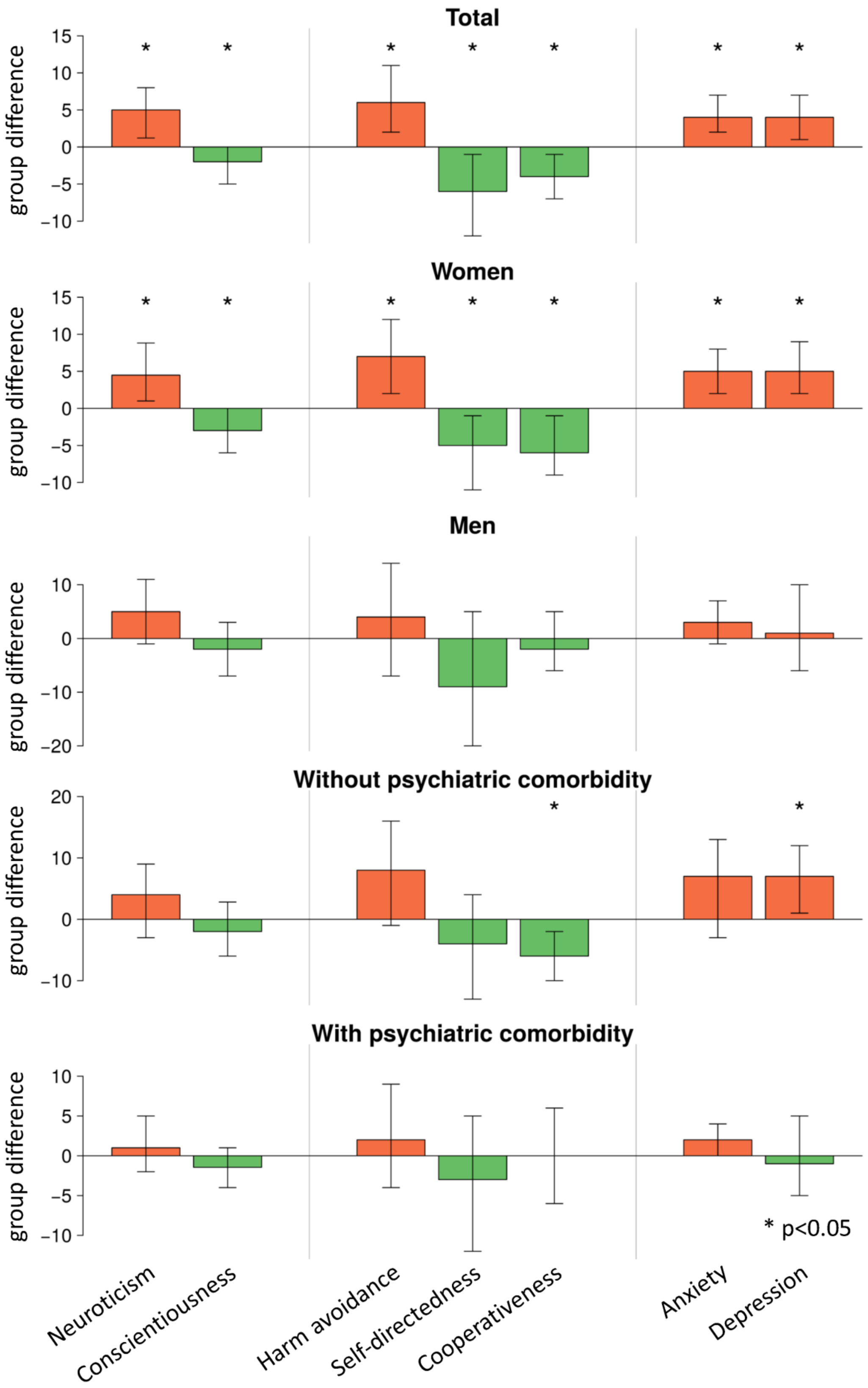

This bar plot presents estimated group differences between MS patients with and without probable PTSD during the SARS-CoV-2 pandemic. The analysis was conducted for the total patient cohort and stratified by sex and the presence of psychiatric comorbidities. For this analysis, the HADS scores (anxiety, depression) were considered alongside the NEO-FFI and TCI-R dimensions that demonstrated significant differences in the main analysis (neuroticism, conscientiousness, harm avoidance, self-directedness, cooperativeness) (Figure 2, Figure 3, Supplementary Table S3). The y-axes show Hodges-Lehmann estimates from Wilcoxon rank-sum tests, with error bars indicating 95% confidence intervals. Red bars indicate higher scores in MS patients with probable PTSD (TSQ score ≥6) compared to those without probable PTSD (TSQ score <6), whereas green bars indicate lower scores in the probable PTSD group. Overall, the subgroup analyses confirmed the associations of personality traits and anxiety/depression scores with pandemic-related trauma. Among the women, all observed differences remained significant (Supplementary Table S4), whereas in other subgroups (men, with psychiatric comorbidity, without psychiatric comorbidity), statistical significance was usually not reached due to smaller sample/effect sizes. HADS, Hospital Anxiety and Depression Scale, MS, multiple sclerosis; NEO-FFI, NEO-Five Factor Inventory; PTSD, post-traumatic stress disorder; SARS-CoV-2, severe acute respiratory syndrome coronavirus 2; TCI-R, Temperament and Character Inventory-Revised; TSQ, Trauma Screening Questionnaire
