# Supplementary material for: Trauma Burden Affected People with Multiple Sclerosis During SARS-CoV-2 Pandemic
Source: J Clin Med. 2025 Apr 13;14(8):2665. doi: 10.3390/jcm14082665 (PMC12027752; doi:10.3390/jcm14082665)
Supplement: Supplementary file 1 [file jcm-14-02665-s001.zip › Supplementary_Figure_S2.pdf]

Supplementary Figure S2. Distribution of personality trait data from the [a] NEO-FFI and [b] TCI-R questionnaires in patients with MS.

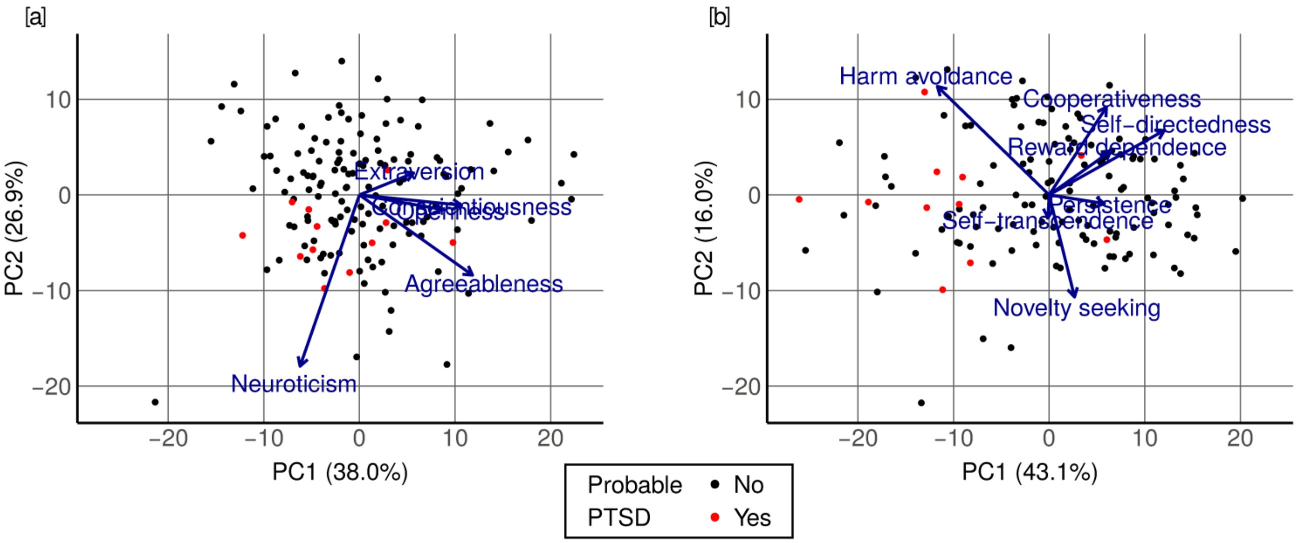

The first two principal components (PC) are displayed in biplots. The blue arrows represent the coefficients of the individual personality dimensions, indicating the direction and strength of their influence on the PCs. These coefficients thus illustrate the relationships between the personality dimensions and the main components of the data. Red points indicate patients with probable PTSD, while black points indicate patients without probable PTSD. MS, multiple sclerosis; NEO-FFI, NEO-Five Factor Inventory; PTSD, post-traumatic stress disorder; TCI-R, Temperament and Character Inventory-Revised
