# Supplementary material for: Trauma Burden Affected People with Multiple Sclerosis During SARS-CoV-2 Pandemic
Source: J Clin Med. 2025 Apr 13;14(8):2665. doi: 10.3390/jcm14082665 (PMC12027752; doi:10.3390/jcm14082665)
Supplement: Supplementary file 1 [file jcm-14-02665-s001.zip › Supplementary_Table_S1.pdf]

Supplementary Table S1. Frequency of responses per item of the adapted TSQ (5-point scale) during the SARS-CoV-2 pandemic among MS patients

Item		˜3 months after COVID-19 outbreak as a global pandemic (*n*=200)	˜1 year after authorization of first SARS-CoV-2 vaccines (*n*=149)
Have any upsetting thoughts or memories about the SARS-CoV-2 pandemic come into your mind against your will? *n* (%)	Not at all	127 (63.5)	94 (63.1)
	Almost not	41 (20.5)	26 (17.4)
	Not sure	15 (7.5)	14 (9.4)
	Strongly	15 (7.5)	13 (8.7)
	Very strongly	2 (1.0)	2 (1.3)
Have you had upsetting dreams because of the SARS-CoV-2 pandemic? *n* (%)	Not at all	180 (90.0)	124 (83.2)
	Almost not	10 (5.0)	13 (8.7)
	Not sure	7 (3.5)	11 (7.4)
	Strongly	3 (1.5)	0 (0.0)
	Very strongly	0 (0.0)	1 (0.7)
Did you act or feel as though this was happening again? *n* (%)	Not at all	143 (71.5)	95 (63.8)
	Almost not	25 (12.5)	15 (10.1)
	Not sure	17 (8.5)	21 (14.1)
	Strongly	12 (6.0)	14 (9.4)
	Very strongly	3 (1.5)	4 (2.7)
Did you feel upset by reminders of the event? *n* (%)	Not at all	131 (65.5)	99 (66.4)
	Almost not	37 (18.5)	18 (12.1)
	Not sure	16 (8.0)	16 (10.7)
	Strongly	14 (7.0)	13 (8.7)
	Very strongly	2 (1.0)	3 (2.0)
Have you experienced bodily reactions (such as fast heartbeat, stomach churning)? *n* (%)	Not at all	155 (77.5)	113 (75.8)
	Almost not	23 (11.5)	15 (10.1)
	Not sure	8 (4.0)	12 (8.1)
	Strongly	11 (5.5)	8 (5.4)
	Very strongly	3 (1.5)	1 (0.7)
Have you experienced difficulties falling or staying asleep? *n* (%)	Not at all	86 (43.0)	88 (59.1)
	Almost not	49 (24.5)	24 (16.1)
	Not sure	24 (12.0)	10 (6.7)
	Strongly	33 (16.5)	21 (14.1)
	Very strongly	8 (4.0)	6 (4.0)
Have you experienced irritability or outbursts of anger? *n* (%)	Not at all	100 (50.0)	88 (59.1)
	Almost not	37 (18.5)	14 (9.4)
	Not sure	18 (9.0)	17 (11.4)
	Strongly	40 (20.0)	25 (16.8)
	Very strongly	5 (2.5)	5 (3.4)
Have you experienced concentration difficulties? *n* (%)	Not at all	84 (42.0)	84 (56.4)
	Almost not	45 (22.5)	18 (12.1)
	Not sure	33 (16.5)	19 (12.8)
	Strongly	29 (14.5)	22 (14.8)
	Very strongly	9 (4.5)	6 (4.0)
Have you dedicated heightened awareness of potential dangers to yourself and others? *n* (%)	Not at all	55 (27.5)	76 (51.0)
	Almost not	46 (23.0)	25 (16.8)
	Not sure	32 (16.0)	12 (8.1)
	Strongly	58 (29.0)	32 (21.5)
	Very strongly	9 (4.5)	4 (2.7)

Did you feel jumpier or were you startled more quickly than usual when something unexpected happened? *n* (%)	Not at all	137 (68.5)	108 (72.5)
	Almost not	41 (20.5)	12 (8.1)
	Not sure	14 (7.0)	9 (6.0)
	Strongly	8 (4.0)	16 (10.7)
	Very strongly	0 (0.0)	4 (2.7)

MS, multiple sclerosis; n, number of patients; SARS-CoV-2, severe acute respiratory syndrome coronavirus type 2; TSQ, Trauma Screening Questionnaire
