# Supplementary material for: Trauma Burden Affected People with Multiple Sclerosis During SARS-CoV-2 Pandemic
Source: J Clin Med. 2025 Apr 13;14(8):2665. doi: 10.3390/jcm14082665 (PMC12027752; doi:10.3390/jcm14082665)
Supplement: Supplementary file 1 [file jcm-14-02665-s001.zip › Supplementary_Table_S2.pdf]

Supplementary Table S2. Frequency of responses after dichotomization per item of the adapted TSQ (2-point scale) and the distribution of TSQ scores during the SARS-CoV-2 pandemic among MS patients

Item		˜3 months after COVID-19 outbreak as a global pandemic (*n*=200)	˜1 year after authorization of first SARS-CoV-2 vaccines (*n*=149)
Have any upsetting thoughts or memories about the SARS-CoV-2 pandemic come into your mind against your will? *n* (%)	Yes	17 (8.5)	15 (10.1)
	No	183 (91.5)	134 (89.9)
Have you had upsetting dreams because of the SARS-CoV-2 pandemic? *n* (%)	Yes	3 (1.5)	1 (0.7)
	No	197 (98.5)	148 (99.3)
Did you act or feel as though this was happening again? *n* (%)	Yes	15 (7.5)	18 (12.1)
	No	185 (92.5)	131 (87.9)
Did you feel upset by reminders of the event? *n* (%)	Yes	16 (8.0)	16 (10.7)
	No	184 (92.0)	133 (89.3)
Have you experienced bodily reactions (such as fast heartbeat, stomach churning)? *n* (%)	Yes	14 (7.0)	9 (6.0)
	No	186 (93.0)	140 (94.0)
Have you experienced difficulties falling or staying asleep? *n* (%)	Yes	41 (20.5)	27 (18.1)
	No	159 (79.5)	122 (81.9)
Have you experienced irritability or outbursts of anger? *n* (%)	Yes	45 (22.5)	30 (20.1)
	No	155 (77.5)	119 (79.9)
Have you experienced concentration difficulties? *n* (%)	Yes	38 (19.0)	28 (18.8)
	No	162 (81.0)	121 (81.2)
Have you dedicated heightened awareness of potential dangers to yourself and others? *n* (%)	Yes	67 (33.5)	36 (24.2)
	No	133 (66.5)	113 (75.8)
Did you feel jumpier or were you startled more quickly than usual when something unexpected happened? *n* (%)	Yes	8 (4.0)	20 (13.4)
	No	192 (96.0)	129 (86.6)
Distribution of TSQ scores, *n* (%)	0-1	137 (68.5)	105 (70.5)
	2-3	48 (24.0)	24 (16.1)
	4-5	6 (3.0)	12 (8.1)
	6-7	8 (4.0)	4 (2.7)
	≥8	1 (0.5)	4 (2.7)
Total TSQ score, median (Q1, Q3; range)		1 (0, 2; 0-8)	0 (0, 2; 0-9)

MS, multiple sclerosis; n, number of patients; Q1, 25% quantile; Q3, 75% quantile; SARS-CoV-2, severe acute respiratory syndrome coronavirus type 2; TSQ, Trauma Screening Questionnaire
