# Supplementary material for: Trauma Burden Affected People with Multiple Sclerosis During SARS-CoV-2 Pandemic
Source: J Clin Med. 2025 Apr 13;14(8):2665. doi: 10.3390/jcm14082665 (PMC12027752; doi:10.3390/jcm14082665)
Supplement: Supplementary file 1 [file jcm-14-02665-s001.zip › Supplementary_Table_S3.pdf]

Supplementary Table S3. Comparison of MS patients with and without probable PTSD regarding NEO-FFI and TCI-R dimensions as well as HADS scores

**Characteristic**	**Total**		**Probable PTSD**		**No probable PTSD**		p^U
	n	median (range)	*n*	median (range)	*n*	median (range)	
NEO-FFI							
Neuroticism	146	22 (9–46)	12	26.5 (18.0–33.8)	134	22 (9–46)	**0.006**
Extraversion	146	24 (5–41)	12	23 (15–33)	134	24.5 (5–41)	0.179
Openness	146	26 (12–45)	12	25.5 (17–45)	134	26 (12–38)	0.705
Agreeableness	146	27 (14–42)	12	28 (24–33)	134	27 (14–42)	0.177
Conscientiousness	146	30 (13–45)	12	28 (21–33)	134	30 (13–45)	**0.030**
TCI-R							
Novelty seeking	133	15 (0–28)	11	14 (6–25)	122	15 (0–28)	0.676
Harm avoidance	133	19 (4–32)	12	27.5 (10–32)	121	18 (4–32)	**0.008**
Reward dependence	134	19 (4–27)	12	18.5 (9–26)	122	19 (4–27)	0.223
Persistence	134	19 (0–31)	12	17 (11–23)	122	20 (0–31)	0.166
Self-directedness	134	30.5 (8–40)	12	25 (8–36)	122	31 (9–40)	**0.022**
Cooperativeness	134	28 (12–35)	12	23 (12–30)	122	28 (12–35)	**0.008**
Self-transcendence	134	6 (1–20)	12	9 (1–18)	122	6 (1–20)	0.286
HADS							
Anxiety	147	7 (0–21)	12	11.5 (1–21)	135	7 (0–17)	**0.001**
Depression	146	6 (0–18)	11	8 (3–17)	135	5 (0–18)	**0.011**

HADS-A, Hospital Anxiety and Depression Scale – Anxiety score; HADS-D, Hospital Anxiety and Depression Scale – Depression score; MS, multiple sclerosis; n, number of patients; NEO-FFI, NEO-Five Factor Inventory; p, p-value; PTSD, post-traumatic stress disorder; TCI-R, Temperament and Character Inventory-Revised; U, Mann-Whitney U test
