# Supplementary material for: Trauma Burden Affected People with Multiple Sclerosis During SARS-CoV-2 Pandemic
Source: J Clin Med. 2025 Apr 13;14(8):2665. doi: 10.3390/jcm14082665 (PMC12027752; doi:10.3390/jcm14082665)
Supplement: Supplementary file 1 [file jcm-14-02665-s001.zip › Supplementary_Table_S4.pdf]

Supplementary Table S4. Comparison of MS patients with and without probable PTSD regarding NEO-FFI and TCI-R dimensions as well as HADS scores, stratified by sex

	**Women**					**Men**				
**Characteristic**	**Probable PTSD**		**No probable PTSD**		p^U	**Probable PTSD**		**No probable PTSD**		p^U
	n	median (range)	*n*	median (range)		*n*	median (range)	*n*	median (range)	
NEO-FFI										
Neuroticism	9	26 (18.0–33.8)	85	21 (9–46)	**0.017**	3	29 (24.0–30.5)	49	22 (14–38)	0.120
Extraversion	9	25 (15–33)	85	24 (5–41)	0.584	3	21 (17–24)	49	25 (17–32)	0.061
Openness	9	25 (17–29)	85	26 (13–37)	0.343	3	31 (17–45)	49	26 (12–38)	0.397
Agreeableness	9	28 (24–32)	85	27 (15–42)	0.138	3	28 (24–33)	49	27 (14–42)	0.844
Conscientiousness	9	28 (21–33)	85	30 (13–45)	**0.037**	3	28 (26–30)	49	30 (20–44)	0.387
TCI-R										
Novelty seeking	8	12 (6–19)	80	14.5 (5–28)	0.081	3	22 (16–25)	42	15 (0–24)	**0.050**
Harm avoidance	9	28 (10–32)	80	18 (5–32)	**0.012**	3	21 (15–29)	41	17 (4–30)	0.351
Reward dependence	9	13 (9–26)	80	20 (5–27)	0.081	3	20 (19–20)	42	16 (4–27)	0.273
Persistence	9	17 (12–23)	80	19.5 (6–31)	0.205	3	18 (11–23)	42	20 (0–30)	0.616
Self-directedness	9	25 (8–36)	80	31 (9–40)	**0.031**	3	16 (12–35)	42	30 (12–40)	0.294
Cooperativeness	9	21 (12–30)	80	28 (12–35)	**0.009**	3	24 (23–28)	42	27 (16–34)	0.423
Self-transcendence	9	6 (1–18)	80	6 (1–18)	0.967	3	13 (12–16)	42	6 (1–20)	**0.028**
HADS										
Anxiety	9	12 (1–21)	86	7 (0–17)	**0.005**	3	9 (8–13)	49	7 (1–15)	0.102
Depression	8	10.5 (5–17)	86	5 (0–18)	**0.005**	3	6 (3–16)	49	6 (0–15)	0.753

HADS-A, Hospital Anxiety and Depression Scale – Anxiety score; HADS-D, Hospital Anxiety and Depression Scale – Depression score; MS, multiple sclerosis; n, number of patients; NEO-FFI, NEO-Five Factor Inventory; p, p-value; PTSD, post-traumatic stress disorder; TCI-R, Temperament and Character Inventory-Revised; U, Mann-Whitney U test
